# Editorial: Optimizing Exercise for the Prevention and Treatment of Type 2 Diabetes

**DOI:** 10.3389/fendo.2018.00237

**Published:** 2018-05-11

**Authors:** Kristian Karstoft, Adeel Safdar, Jonathan P. Little

**Affiliations:** ^1^The Centre of Inflammation and Metabolism, The Centre for Physical Activity Research, Rigshospitalet, University of Copenhagen, Copenhagen, Denmark; ^2^Department of Clinical Pharmacology, Bispebjerg Hospital, University of Copenhagen, Copenhagen, Denmark; ^3^School of Health Sciences, Humber College, Toronto, ON, Canada; ^4^School of Health and Exercise Sciences, University of British Columbia, Kelowna, BC, Canada

**Keywords:** exercise, diabetes mellitus, type 2, motivation, lifestyle interventions, prediabetes

**Editorial on the Research Topic**

**Optimizing Exercise for the Prevention and Treatment of Type 2 Diabetes**

## Introduction

That exercise is beneficial for the prevention and treatment of type 2 diabetes (T2D) is not a novelty, and exercise is indeed regarded as a front-line therapy in T2D (1). In recent years, increasing attention has focused on how to manipulate the exercise stimulus to optimize beneficial responses. As such, factors, including intensity, volume, timing, and potential interactions with diet and medication have each, and in various combinations, been suggested to play pivotal roles in exercise efficacy. Despite this encouraging research, the optimal exercise strategy is far from determined, which is why this Research Topic was introduced.

This Research Topic consists of 10 articles, of which five contain original data and five are review/opinion articles. A broad range of themes are covered, ranging from clinical effects of different types of exercise, to mechanisms underlying exercise-induced improvements in metabolic markers, and expanding to perspectives on why exercise may be important for hard endpoints and how motivation toward physical activity may be regulated.

## Interval Training Modalities

Interval training, especially high-intensity interval training (HIIT), has in the recent years gained momentum in prevention and treatment of T2D. As a result, HIIT was recently—for the first time—included in the ADA position stand about physical activity/exercise and diabetes (2), as outlined by Colberg, with HIIT now being recommended as an alternative approach to continuous aerobic exercise for some individuals with diabetes. Since some researchers have argued that the inclusion of HIIT in the treatment of metabolic diseases is premature given that only few and small studies exist in relevant populations (3), it is of high interest that several large HIIT studies are included in this Research Topic (Phillips et al.; Francois et al.; Alvarez et al.). This includes the largest published HIIT trial, to our knowledge, in individuals with prediabetes [*N* = 189 (Phillips et al.)]. Overall, these studies suggest that supervised HIIT robustly improves glycemic control and other cardiovascular risk factors in individuals with or at risk for T2D. In contrast, HIIT does not seem to affect basic metabolic rate (Karstoft et al.). All together, these studies report results from *N* = 304 individuals undergoing HIIT, advancing the notion that HIIT is a feasible and effective training strategy, also in participants with metabolic disease.

## Mechanisms

Bearing the above-standing beneficial effects of HIIT in mind, and also acknowledging that HIIT may be superior to moderate-intensity continuous training (4–6), it is of interest to assess which mechanisms that are responsible for the improvements in cardiovascular risk factors seen with HIIT. In this context, several insightful articles are included in the Research Topic. As reviewed by Carson, myokines are proteins that are released by muscles and have auto-, para-, and/or endocrine functions; some of which are known to affect cardiovascular risk factors. Several of the known myokines are induced by contraction, and given that this induction is dependent on exercise intensity (7), it is intriguing to speculate that some of the effects of HIIT are mediated *via* contraction-induced myokines. Also relevant in this context, Eshghi et al. showed that the timing of exercise may be important, since exercise-induced increase in systemic levels of the myokine IL-6 is only seen following the first of two similar exercise bouts performed at one single day. The idea of so-called “non-response” or individualized responses to exercise training is a hot, yet somewhat controversial, topic in the field (8). This was addressed in a preliminary report from Alvarez et al., which suggested that baseline insulin resistance might influence certain cardiometabolic responses to HIIT in women.

In a comprehensive review, Parker et al. reviewed the complex interplay between oxidative stress, antioxidant defense, and physical activity. Whereas both inactivity/obesity on one side and acute exercise on the other side results in increased systemic levels of oxidative stress, the effects on glycemic control and insulin sensitivity are opposing. Parker et al. suggests that differences in intracellular signaling and antioxidant defense may be responsible for these discrepancies. Again, given that the effect of exercise on oxidative stress is dependent on exercise intensity (9), it may be speculated that some of the improvements seen with HIIT are dependent on changes in oxidative stress and antioxidant defense.

## New Insights

Whereas the HIIT-induced improvements in cardiovascular risk factors are interesting, it must be acknowledged that little is known about the effects of HIIT (and other types of exercise) on hard endpoints. Given that high postprandial glucose excursions are suggested to be more deleterious than elevated mean blood glucose levels for cardiovascular risk factors (10, 11), and since postprandial exercise is known to effectively reduce glucose excursions (12), Erickson et al. suggest that exercise for T2D subjects should in general be prescribed post-meal and individualized according to the need, with large glucose excursions requiring longer and more intense exercise bouts compared to small glucose excursions.

For benefits of any type of exercise, the need to adhere is fundamental. The review by Ruegsegger and Booth provides exciting new insights into the importance of the mesolimbic system in controlling motivation and physical activity behavior *via* dopaminergic signaling. Understanding these processes is imperative if the general trend in the population, where physical activity levels are decreasing, is to be reversed.

## Perspectives

Papers in this Research Topic highlight that exercise has a role in the prevention and treatment of T2D. Whereas HIIT seems to be effective for improving cardiovascular risk factors, we still need to characterize the mechanisms underlying the improvements seen in order to develop even more effective training programs for individuals with or at risk for T2D. Moreover, whereas efficacy of supervised HIIT is evident, effectiveness of unsupervised “real-life” HIIT is largely unknown and limited to small studies (13, 14). In order for HIIT and other novel types of exercise to be implemented clinically, more work is needed. Interdisciplinary research involving mechanisms like myokines, oxidative stress, and brain reward systems coupled with innovative real-world trials of HIIT and traditional exercise seem an exciting avenue for optimizing exercise for the prevention and treatment of T2D.

## Author Contributions

KK and JL drafted the manuscript with input from AS. All authors approved the final version.

## Conflict of Interest Statement

The authors declare that the research was conducted in the absence of any commercial or financial relationships that could be construed as a potential conflict of interest.

## References

[1] American Diabetes Assocaition. Lifestyle management Sec. 4 in standards of medical care in diabetes – 2017. Diabetes Care (2017) 40:S33–43. 10.2337/dc17-S007 27979891

[2] Colberg SR Sigal RJ Yardley JE Riddell MC Dunstan DW Dempsey PC Physical activity/exercise and diabetes: a position statement of the american diabetes association. Diabetes Care (2016) 39:2065–79. 10.2337/dc16-1728 27926890 PMC6908414

[3] Holloway TM Spriet LL CrossTalk opposing view: high intensity interval training does not have a role in risk reduction or treatment of disease. J Physiol (2015) 593:5219–21. 10.1113/JP271039 26641011 PMC4704522

[4] Tjonna AE Lee SJ Rognmo O Stolen TO Bye A Haram PM Aerobic interval training versus continuous moderate exercise as a treatment for the metabolic syndrome: a pilot study. Circulation (2008) 118:346–54. 10.1161/CIRCULATIONAHA.108.772822 18606913 PMC2777731

[5] Karstoft K Clark MA Jakobsen I Muller IA Pedersen BK Solomon TP The effects of 2 weeks of interval vs continuous walking training on glycaemic control and whole-body oxidative stress in individuals with type 2 diabetes: a controlled, randomised, crossover trial. Diabetologia (2017) 60:508–17. 10.1007/s00125-017-4406-0 27942800

[6] Little JP Jung ME Wright AE Wright W Manders RJ. Effects of high-intensity interval exercise versus continuous moderate-intensity exercise on postprandial glycemic control assessed by continuous glucose monitoring in obese adults. Appl Physiol Nutr Metab (2014) 39:835–41. 10.1139/apnm-2013-0512 24773254

[7] Helge JW Stallknecht B Pedersen BK Galbo H Kiens B Richter EA. The effect of graded exercise on IL-6 release and glucose uptake in human skeletal muscle. J Physiol (2003) 546:299–305. 10.1113/jphysiol.2002.030437 12509497 PMC2342463

[8] Atkinson G Batterham AM. True and false interindividual differences in the physiological response to an intervention. Exp Physiol (2015) 100:577–88. 10.1113/EP085070 25823596

[9] Fisher-Wellman K Bell HK Bloomer RJ. Oxidative stress and antioxidant defense mechanisms linked to exercise during cardiopulmonary and metabolic disorders. Oxid Med Cell Longev (2009) 2:43–51. 10.4161/oxim.2.1.7732 20046644 PMC2763230

[10] DECODE Study Group, European Diabetes Epidemiology Group. Is the current definition for diabetes relevant to mortality risk from all causes and cardiovascular and noncardiovascular diseases? Diabetes Care (2003) 26:688–96. 10.2337/diacare.26.3.688 12610023

[11] Sasso FC Carbonara O Nasti R Campana B Marfella R Torella M Glucose metabolism and coronary heart disease in patients with normal glucose tolerance. JAMA (2004) 291:1857–63. 10.1001/jama.291.15.1857 15100204

[12] Reynolds AN Mann JI Williams S Venn BJ. Advice to walk after meals is more effective for lowering postprandial glycaemia in type 2 diabetes mellitus than advice that does not specify timing: a randomised crossover study. Diabetologia (2016) 59:2572–8. 10.1007/s00125-016-4085-2 27747394

[13] Jung ME Bourne JE Beauchamp MR Robinson E Little JP. High-intensity interval training as an efficacious alternative to moderate-intensity continuous training for adults with prediabetes. J Diabetes Res (2015) 191595:2015. 10.1155/2015/191595 25918728 PMC4396724

[14] Karstoft K Winding K Knudsen SH Nielsen JS Thomsen C Pedersen BK The effects of free-living interval-walking training on glycemic control, body composition, and physical fitness in type 2 diabetes patients. Diabetes Care (2013) 36:228–36. 10.2337/dc12-0658 23002086 PMC3554285

